# Improved Methods of Treatment in Deformities

**Published:** 1863-12

**Authors:** E. Andrews

**Affiliations:** Professor of Surgery in Chicago Medical College


### IMPROVED METHODS OF TREATMENT IN DEFORMITIES.

By E. ANDREWS, A.M., M.D., Professor of Surgery in Chicago Medical College.

In previous articles, I have figured and described several pieces of apparatus, which I use for the cure of spinal curvatures. There are numerous other appliances which are valuable adjuvants to the main treatment, among the most important of which is what the German surgeons call the "stretch-bed." This machine consists of a couch with various appliances at the head and foot for making extension and counter-extension upon the spinal column; by means of which, like a string put under tension, the curves of the spinal column are drawn gradually straight. The first successes obtained by this invention produced quite a furor in its favor in Europe; and its popularity was such as to occasion the satire, that "many seemed to imagine that nothing more was necessary to constitute an orthopedic surgeon than a stretch-bed and patients."

The extending power in this machine consists of springs or weights and pulleys, applied both at the head and the foot of the bed, the weights being preferable. The upper extenders were applied to the head, in case of high curvature, and to the shoulders when the difficulty was lower down. The lower extension was applied to the bulge of the hips or to the feet. The patient was not usually required to remain continuously in the machine, but was placed in it, at intervals, from two to four times a-day; but the more time he could spend in it, without injurious loss of exercise, the more rapid was his improvement.

Although the extravagant admiration at first felt for the stretch-bed has greatly subsided, it still remains as a very valuable instrument, which no one undertaking the treatment of spinal curvatures can afford to do without. It may be specially and elaborately constructed for hospital purposes, or be extemporized out of an ordinary bedstead in private practice. For hospital purposes, the bedstead may be made of wood or iron. It should be not less than eight feet in length, by three and a-half in breadth. The great length is required to make space above the head, and below the feet for elastic straps and other extending appliances. There should be no head nor foot board, but instead of them a long roller of wood, three inches in diameter, extending from post to post, across the head and foot of the machine, and turning easily on iron axles. Above each roller should be a strong crossbar of wood, into which iron pullies may be set in various positions, as the surgeon may from time to time desire. The mattress should be of curled hair, rather hard, and made level and smooth. Pillows and bolsters can be varied according to the necessities of the case.

For temporary use, in private practice, a stretch-bed may be improvised out of the ordinary bedstead, by cutting openings through the head and foot boards, and setting in some small cast-iron or brass pulleys, such as may be found in any hardware store.

If the deformity is in the upper portion of the spine, an extension is attached to the head, by means of a firm leather band, moulded to the occupit, and provided with two branch straps, one to cross the forehead, and the other to pass under the point of the chin. This must be very carefully constructed, or else it will be too irksome to be borne, but when well fitted, it is borne without pain. A short band passes upward from each side of the head, and attaches to a cord which is passed over the pulley and supports a weight. The counter-extension is made by a cord and weight at the foot of the bed, in a similar manner, and may be attached to the patient either by adhesive straps applied to the legs, or by a strong waist buckled around the bulge of the hips. The weights should vary from five pounds upward, according to the ability of the patient to tolerate it. If this apparatus is properly constructed and applied, the patient will enjoy free motion both of upper and lower extremities, and can turn on his back, his face, or either side, without interfering with the extension, or rising from the bed. No effort should be made to keep the patient continually on the stretch-bed, except in cases where he is unable to sit or walk. He should resort to the bed from two to four times a-day, and remain from half an hour to an hour and a-half each time. The remainder of the time he should either wear a proper supporter—such as I have described in a former article,—or be occupied by gymnastic exercises calculated to correct the deformity. Some patients will be able to sleep in the stretch-bed after a little practice. In these cases they should by all means do so, as it adds the whole of the sleeping hours to the treatment, and very much hastens the recovery. If the deformity is below the sixth dorsal vertebra, the upper extension should be applied to the armpits and chest by proper pads in the axilla, and by broad adhesive straps upon the back and chest, attached to the extending cord. When properly used, the stretch-bed exerts a very powerful influence in unfolding spinal curvatures, and the worse the deformity the more striking are its results. One of the most prominent symptoms of improvement is the surprising increase of stature which the patient exhibits as the spinal column comes out to a correct line.

GYMNASTICS.

The cure of some forms of curvature of the spine, and of all anchylosed joints, is greatly promoted, and may be entirely accomplished, by proper specific exercises, either active or passive. It is almost impossible to introduce this part of the treatment fully into general practice, on account of the amount of time required to be spent with the patient, either by the surgeon, or by a trained assistant, but parts of it will be found useful to every practitioner. The exercises are active and passive, the former being executed by the patient's own muscles, and the latter by the hand of the surgeon. Thus, for instance, if the patient has a slight double lateral curvature, and he be directed to elevate the shoulder on the side of the concavity of the upper curve (usually the left,) and depress the opposite one, and to curve the spine in the direction opposite the deformity, the practitioner at the same time guiding and assisting the motion with the hands, it will be found that the spine is momentarily restored to its normal shape. If she now repeat these motions with the same assistance many times, until fatigued, every day, the muscles which are thus trained will acquire a prodigious development, and their antagonists remaining undeveloped, they gain the mastery, and by their own superior tension ultimately correct the deformity. The bones and ligaments yield slowly to the pressure, until their shapes are perfectly restored. This is the principle of Ling's Swedish "Movement Cure," which, in a debased and spoiled form, is now hawked about the country, by sundry quacks. Some additional exercises are performed in most cases. Thus a cushioned post is prepared, and set firmly in the floor, across the top of which the patient is made to lean, and by repeated efforts of the surgeon, is made many times in succession to flex the curved spine, in the direction opposite that of the deformity.

Anchylosed joints are treated by constantly repeated exercises, both active and passive, until by degrees the fibrous bands are elongated, and mobility established. A vast number of other exercises have been devised by various orthopedists, some of which are useful and some not, but the principles involved are the same throughout.

### STATE OF ORTHOPEDIC SURGERY IN EUROPE.

Dr. Ling, of Stockholm, was one of the earliest lights in orthopedy. His system of treatment consisted mainly in the series of gymnastic exercises alluded to above. He gave a strong impetus to the treatment of deformities; and his institute was under the patronage of the government for forty years. Sundry rags and tatters of his ideas, under the name of the "Swedish Movement Cure," constitute the stock in trade of numerous American quacks.

Wildberger, of the Orthopedic Institute in Bamberg, mostly discards Ling's gymnastics as useless, because they are very unsuccessful in spinal diseases. This is, in a great measure, true, Ling's exercises being better adapted to diseases of the extremities than of the spine. Wildberger, on the contrary, gives most of his attention to spinal deformities, and treats them mainly by a variety of splints and supporters, which slowly and steadily force the curvatures back to a straight line. His apparatus is thorough and efficient in its action, and has the merit of allowing the patient to walk about and exercise while it is worn; but most of it is complex and clumsy in structure, being in striking contrast, in that respect, with American instruments.

Dr. Melicher, of Vienna, has an orthopedic institute, in which he does, or at least did, a few years ago, rely almost exclusively upon Ling's gymnastics.

Dr. Berend, of Berlin, has an establishment in which he treats his patients by tenotomy, or other surgical operation, when required, and by the stretch-bed and other machinery, after which he completes the cures by gymnastics alone.

Dr. Schreber, of the Leipsic Orthopedic Institute, treats his patients upon a stretch-bed, of which the extending force is produced by steel springs. The bed is also provided with lateral steel springs, to press in the convexities of the curved spines. His institute is but little patronized.

Dr. Kjœlstadt, of Norway, has complex system something like the following. He first places his patient upon a stretch-bed, during certain hours. Then taking him up, he places him in a peculiar machine, in which he marches him with short steps around the room. Then laying him down, he kneads the joints and muscles with his fists, and then returns him to the stretch-bed again. He is said to possess very little adaptive power, treating all kinds of cases alike.

Dr. ROTH, of London, has an institute, in which he follows Ling's method, combined with the Russian bath,—that is a bath having a series of sudden alternations between hot and cold water.

Dr. NITZSCHE, of Dresden, takes complete possession of his patients, occupying their whole time with curative measures, making extensive use of gymnastics and electricity. Spinal curvatures go through the following course:—In the morning, he first washes the patient's back with cold water; then laying him on his face, he rubs him down with alcohol, and proceeds to knead and press the back in a systematic manner. He then practices the sufferer on motions to straighten the spine by the action of his own muscles. Next comes a series of exercises in which the spine is stretched between rollers, and the patient is made to swing by his hands, head, &c., &c. All this is the morning lesson. In the afternoon it is repeated, and the evening is occupied with gymnastics; after which his patients are said to sleep well. If they are not cured it certainly is not for want of diligence.

Dr. KLEPSCH, of the Breslau Institute, uses stretch-beds, electricity, and a variety of instruments for club-feet, and other deformities.

Dr. KNORR, of Munich, takes substantially the same course, adding to it, however, a system of gymnastics and of water cure.

Dr. PARROW, of the Orthopedic Institute, in Bonn, has a kind of chair constructed for straightening the spine. He also makes use of a great variety of apparatus, among which are pulleys, springs, and sundry handles pendent from the ceiling, upon which the patient practices swinging by one or both hands, as the case requires.

Drs. EBENER & GROSSMAN, of Stutgard, regard instruments as indispensible, employing stretch-beds, corsets, supporters, &c., and adding also active and passive gymnastics.

Prof. Werner, of the Gymnastic Academy, of Dessau, employs corsets, supporters, stretch-beds, and baths, together with active gymnastics, but condemns the passive gymnastics as useless. In this, however, he is certainly in error, as the passive movements are very often the only ones which are possible at the commencement of the treatment.

STATE OF ORTHOPEDIC SURGERY IN THE UNITED STATES.

In this country, the cure of deformities is an almost completely neglected art. A few good men are zealously cultivating it in the larger Eastern cities; but in the West, it has only just begun to receive attention. For this reason, the whole country is filled with neglected spinal curvatures, bent knees, uncured club-feet, and anchylosed elbows. Many of these cases are perfectly curable, even when of many years' standing, and should be at once taken in hand. The cases of spinal deformity are especially to be commiserated, because they are usually taught to look upon their state as hopeless; whereas, a large portion of them are capable of being restored to soundness and perfect form. It is the hope of the writer, that these articles may arouse the attention of our surgeons to their duty; and prevent these cases from being turned over to the maltreatment of lying, itinerant quacks.

---

Gonorrhœal Ophthalmia.—This formidable affection yields with marvellous rapidity to repeated weak injections. In the most acute cases a solution of a-quarter of a grain of nitrate of silver to the ounce of distilled water should be injected beneath the upper lid, with a syringe, every ten minutes, for the first hour; after that, a-half grain solution should be injected every half-hour. If this is carefully carried out for the first 24 hours, the patient's eye will be quite safe. A stronger solution may then be used, but less frequently, and in a couple of days, if the villous condition of the conjunctiva should seem to require it, Guthrie's ointment of nitrate of silver may be used. These weak solutions are quite useless unless very frequently applied; but if so used, scarcely an eye need be lost from gonorrhœal ophthalmia. (Dr. M. H. Collins, page 183.)

